# Novel Loss of Function Variants in *CENPF* Including a Large Intragenic Deletion in Patients with Strømme Syndrome

**DOI:** 10.3390/genes14111985

**Published:** 2023-10-24

**Authors:** Doriana Misceo, Lokuliyanage Dona Samudita Senaratne, Inger-Lise Mero, Arvind Y. M. Sundaram, Pål Marius Bjørnstad, Krzysztof Szczałuba, Piotr Gasperowicz, Benjamin Kamien, Bård Nedregaard, Asbjørn Holmgren, Petter Strømme, Eirik Frengen

**Affiliations:** 1 Department of Medical Genetics, Oslo University Hospital, 0450 Oslo, Norway; doriana.misceo@medisin.uio.no (D.M.); ssamudita@gmail.com (L.D.S.S.); uxwmin@ous-hf.no (I.-L.M.); arvind.sundaram@medisin.uio.no (A.Y.M.S.); asbjorn.holmgren@medisin.uio.no (A.H.); 2 Faculty of Medicine, University of Oslo, 0450 Oslo, Norway; petter.stromme@medisin.uio.no; 3 Department of Medical Genetics, Medical University of Warsaw, Żwirki i Wigury 61, 02-091 Warszawa, Poland; krzysztof.szczaluba@gmail.com (K.S.);; 4 Genetic Services of Western Australia, King Edward Memorial Hospital, 374 Bagot Rd, Subiaco, WA 6008, Australia; benjamin.kamien@health.wa.gov.au; 5 Department of Radiology and Nuclear Medicine, Section of Neuroradiology, Oslo University Hospital, 0450 Oslo, Norway; bnedrega@ous-hf.no; 6 Division of Pediatric and Adolescent Medicine, Oslo University Hospital, 0450 Oslo, Norway

**Keywords:** *CENPF*, ciliopathy, intestinal atresia, LR-WGS, structural variant, Strømme syndrome

## Abstract

Strømme syndrome is an ultra-rare primary ciliopathy with clinical variability. The syndrome is caused by bi-allelic variants in CENPF, a protein with key roles in both chromosomal segregation and ciliogenesis. We report three unrelated patients with Strømme syndrome and, using high-throughput sequencing approaches, we identified novel pathogenic variants in *CENPF*, including one structural variant, giving a genetic diagnosis to the patients. Patient 1 was a premature baby who died at 26 days with congenital malformations affecting many organs including the brain, eyes, and intestine. She was homozygous for a donor splice variant in *CENPF*, NM_016343.3:c.1068+1G>A, causing skipping of exon 7, resulting in a frameshift. Patient 2 was a female with intestinal atresia, microcephaly, and a Peters anomaly. She had normal developmental milestones at the age of 7 years. She is compound heterozygous for *CENPF* NM_016343.3:c.5920dup and c.8991del, both frameshift. Patient 3 was a male with anomalies of the brain, eye, intestine, and kidneys. He was compound heterozygous for CENPF p.(Glu298Ter), and a 5323 bp deletion covering exon 1. *CENPF* exon 1 is flanked by repetitive sequences that may represent a site of a recurrent structural variation, which should be a focus in patients with Strømme syndrome of unknown etiology.

## 1. Introduction

The clinical entity of Strømme syndrome (OMIM 243605) was described in 1993 by Strømme et al. in two siblings with apple-peel jejunal atresia (type IIIb), ocular anomalies, microcephaly, and short stature [1,2]. Later, additional clinical manifestations were recognized as part of this syndrome, such as prematurity, developmental delay/intellectual disability, cranio-facial dysmorphisms, cerebral, pulmonary, skeletal, and genitourinary malformations [1,2,3,4,5,6,7,8,9,10,11,12,13,14,15]. The clinical severity is highly variable, ranging from microcephaly with or without learning disabilities to multiorgan abnormalities causing lethality at mid-gestation or in infancy [1,2,3,4,5,6,7,8,9,10,11,12,13,14,15]. In 2015, *CENPF* null alleles were shown to cause Strømme syndrome (OMIM 243605) with autosomal recessive inheritance [15]. *CENPF* encodes for Centromeric protein F, a large microtubule regulating protein, dynamically expressed throughout the cell cycle [16]. Depending on the stage of the cell cycle, CENPF localizes in different intracellular sites, including the nuclear envelope and kinetochore, where it has a central role in chromosomal segregation [17,18]. In addition, CENPF localizes at the cilium, both at the sub-distal appendages of the basal body and in the axoneme, and it has a role in cilia biology [15]. Based on CENPF’s intracellular localization and on the presence of ciliopathy phenotypes in zebrafish *cenpf* morphants, Strømme syndrome was concluded to be a primary ciliopathy [15].

Strømme syndrome is ultra-rare, with 21 patients and five fetuses from a total of 18 families reported in literature [1,2,3,4,5,6,7,8,9,10,11,12,13,14,15]. Many of the patients with Strømme syndrome reported in the literature are Caucasian or from Middle East [1,3,4,5,6,7,8,9,12,13,14,15,19]. However, this might not be a real population-based difference in the prevalence of Strømme syndrome, but rather be due to ascertainment bias, as many studies have focused on European and Middle Eastern populations. We describe three unrelated patients with clinical features of Strømme syndrome and bi-allelic variants in *CENPF*. Patient 1 was a premature female born with neurological, ophthalmological, intestinal, and renal anomalies, who died at 26 days. She harbored a homozygous variant in *CENPF* NM_016343.3:c.1068+1G>A, p.(Glu289Valfs*33). Patient 2, a female patient primarily affected by intestinal atresia, was compound heterozygous for *CENPF* NM_016343.3:c.5920dup, p.(Thr1974Asnfs*9) and NM_016343.3:c.8991del, p.(Ser2998Alafs*23), both causing frameshifts. Patient 3 was a male patient born with ocular and intestinal anomalies. He was compound heterozygous for a frameshift variant resulting in a premature stop codon *CENPF* NM_016343.3:c.892G>T, p.(Glu298Ter), and for a 5323 bp intragenic deletion of exon 1 and part of intron 1. This is the first report of a large intragenic deletion in a patient with Strømme syndrome that we identified by long reads-whole genome sequencing (LR-WGS). The deleted genomic region in patient 3 overlapping *CENPF* exon 1 may be a site of a recurrent structural variation and should be investigated carefully in patients with a clinical diagnosis of Strømme syndrome.

## 2. Materials and Methods

### 2.1. Whole Exome Sequencing (WES) and Variant Verification

In patient 1 and 3, Whole Exome Sequencing (WES) was performed on genomic DNA isolated from peripheral blood. Samples prepared using the SureSelectXT Human All Exon v5 (Agilent Technologies, Santa Clara, CA, USA) were sequenced on Illumina HiSeq2000 instrument (Illumina Inc., San Diego, CA, USA), and WES data were analyzed as previously described [20]. Sanger sequencing was performed on DNA from patient 1 and parents using the following primers: forward GAACTACGCCTGCAAGGACA and reverse TGGGAAAGTACGGCACCAG. In patient 3, Sanger sequencing was performed using the following primer pair: forward GTTTGTGGTTCAAGAGCTAAGAAAC, reverse ATTCACGAAAAGTCAAGTACCTTGG.

Blood for RNA extraction was collected from the parents in family 1 using PAX Blood RNA Tubes, and RNA extracted with PAXgene Blood RNA Kit (PreAnalytiX GmbH, Hombrechtikon, Switzerland). RNA to cDNA conversion was carried out with High Capacity cDNA Reverse Transcription Kit (Thermo Fisher Scientific, Waltham, MA, USA). The following primers were used on cDNA: forward ATGGCAGCAAGAGAAGACC and reverse AGGCTGAACTGGATAAACTCACAT.

Patient 2 had a clinical diagnosis of Strømme syndrome; therefore, only the *CENPF* gene was interrogated by custom NGS testing (www.fulgentgenetics.com/).

### 2.2. RNA Sequencing (RNA-seq) and Data Analysis

RNA-seq was performed on RNA extracted from skin-derived fibroblasts cultured in Dulbecco’s modified Eagle’s medium (DMEM) (Life Technologies, Carlsbad, CA, USA) supplemented with 10% (*v*/*v*) fetal bovine serum and 100 U/mL penicillin and 100 μg/mL streptomycin. Cells were cultured at 37 °C in 5% CO_2_. RNA was extracted using Ambion PARIS™ system (Thermo Fisher Scientific). Six samples, three controls, and a biological triplicate of cell from patient 3 were prepared for RNA-seq with the Illumina Strand-specific TruSeq mRNA-seq library prep (Illumina Inc.). The libraries were indexed, pooled, and sequenced on an Illumina NovaSeq SP (Illumina Inc.) with 150 bp paired end reads. Reads were aligned to the ENSEMBL reference GRCh37 release87 (Homo_sapiens.GRCh37.DNA.primary_assembly.fa and Homo_sapiens.GRCh37.87.gtf) with HISAT2 2.1.0 [21]. The analysis was performed using the R software R 4.1.2 [22], Bioconductor [23] packages including DESeq2 1.22.1 [24,25] and the SARTools 1.6.8 [26]. Normalization, in order to make gene read counts comparable across samples and to perform differential analysis, was carried out according to the DESeq2 model and package.

### 2.3. Long-Read Whole Genome Sequencing

High-molecular weight genomic DNA from patient 3 and three controls were extracted from skin fibroblasts cultivated as described above using Circulomics Nanobind CBB Big DNA Kit (Circulomics, Baltimore, MD, USA). Library was prepared using Pacific Biosciences protocol for HiFi library prep using SMRTbell^®^ ExpressTemplate Prep Kit 2.0 (PacBio, Menlo Park, CA, USA). DNA was fragmented to 15–20 kb fragments using Megaruptor 3 (Diagenode s.a, Liège, Belgium). Final library was size selected using BluePippin (Sage Science, Inc., Beverly, MA, USA) with 10 kb cut-off. The library was sequenced on one 8M SMRT cell on Sequel II instrument using Sequel II Binding kit 2.2 and Sequencing chemistry v2.0. Loading was performed by adaptive loading, movie time: 30 h. Sequencing was conducted in circular consensus sequencing (CCS) mode and CCS sequences generated using CCS pipeline (SMRT Link v10.2.0.133434). Reads with at least 99% accuracy are called High Fidelity (HiFi) reads and were delivered in separate file. The reads were aligned to the Human reference genome (GRCh38) using pbmm2 v1.5 and variants were called with pbsv v2.4.1 (both pbmm2 and pbsv are part of the SMRT Link package). The generated bam files were analyzed using Integrative Genomics Viewer 2.14.1 (IGV) [27].

## 3. Results

### 3.1. Clinical Presentations

An overview of the genetic and clinical findings is presented in Table 1.

**Figure 1:**
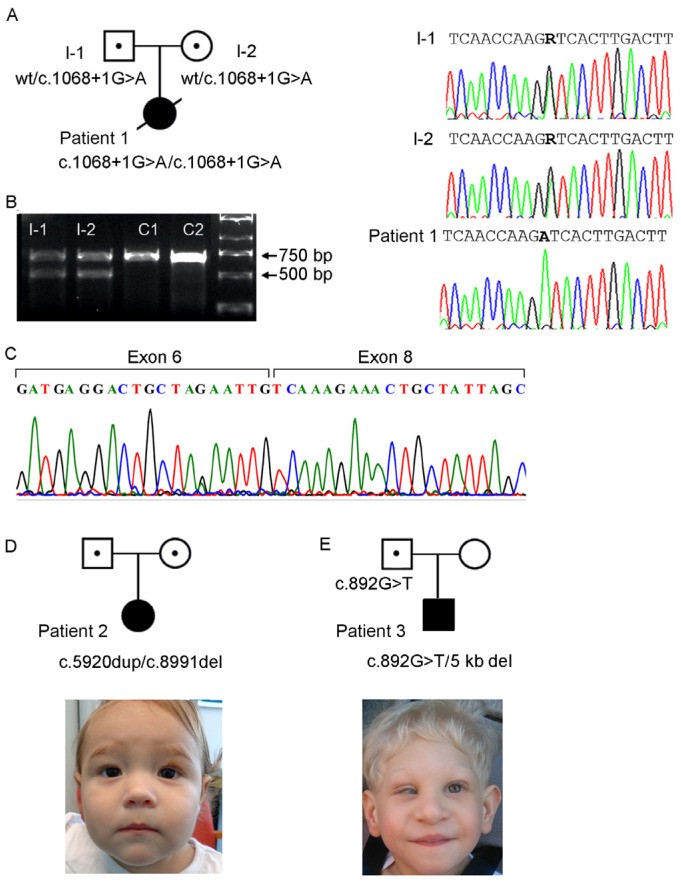
(**A**) Pedigree of patient 1 and segregation of the *CENPF* variant indicated in bold. R = indicates A or G. (**B**) rtPCR products from leukocyte RNA from the parents of patient 1 (I-1 and I-2) and two controls (C1 and C2). Primers located in *CENPF* exon 6 (forward) and exon 9–10 (reverse), detected the expected 678 bp PCR product in both parents and controls. In addition, a second smaller band was detected in the parents. (**C**) Sanger sequencing of the smaller PCR products shown in (**B**) documented skipping of *CENPF* exon 7 (203 bp) in the parents of patient 1 (I-I and I-2). (**D**) Pedigree of patient 2 with *CENPF* variants and underneath photo of patient 2 age 15 months showing short palpebral fissures and sparse eyebrows, particularly at the medial aspects. Palpebral fissures are mildly upslanting. (**E**) Pedigree of patient 3 with segregation of the *CENPF* variants and photo at the age of 2 years. Note the right eye microphthalmia and sclerocornea.

**Figure 2:**
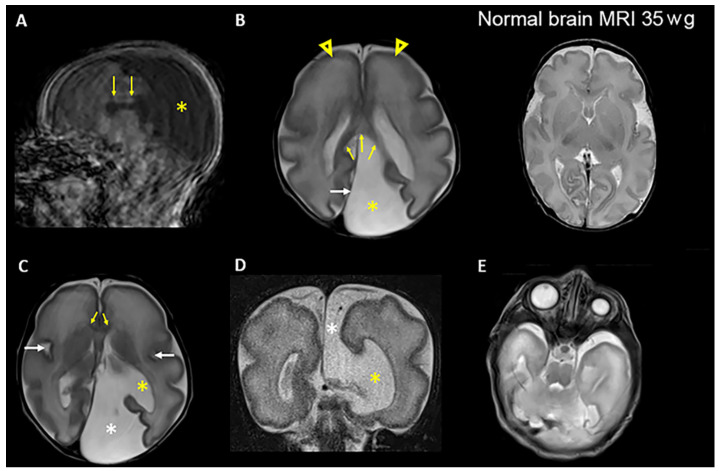
Brain MRI images of patient 1 in the postnatal period at approximately 35 weeks gestational age demonstrate multiple congenital anomalies. (**A**) Sagittal T1-weighted image shows the presence of the corpus callosum, which is not properly developed (arrows). The posterior portion (the splenuim) is located further anterior than normal. CSF fills most of the posterior supratentorial compartment (asterisk). Note motion artifacts in the basal regions of the brain. (**B**) Left: Axial T2-weighted image shows an expansion of the left supratentorial cranial cavity (interhemispheric cyst) filled with CSF (asterisk) in the parietooccipital region of the left hemisphere. The posterior falx cerebri (white arrow) is shifted toward the right hemisphere. The splenium of the corpus callosum is visible (yellow arrows). There is primitive gyrosulcal patterning, more prominent in the frontal lobes (arrowheads), consistent with 28–29 weeks of gestation. Right: Axial T2-weighted image shows normal gyrus and sulcus formation at 35 weeks of gestation (wg). (**C**) Axial T2-weighted image shows the interhemispheric cyst (white asterisk) communicating with the enlarged left lateral ventricle (yellow asterisk). The genu of the corpus callosum is visible (yellow arrows). The sylvian fissures (white arrows) appear underdeveloped in both hemispheres. (**D**) Coronal T2- weighted image shows the communication of the CSF space (interhemispheric cyst) (white asterisk) with the lateral ventricle (yellow asterisk) on the left side through a broad parenchymal defect in the medial temporoparietal region. (**E**) Axial T2-weighted image shows pronounced left microphthalmia, without detectable lens.

Patient 1 was a female only child, born at 34 + 3 weeks to a healthy consanguineous Lithuanian couple (Figure 1A). She was small for gestational age with weight 1360 g (<3rd centile), length 39 cm (3rd centile) and OFC 25.8 cm (3 cm < 3rd centile). Fetal ultrasound examination showed cerebral ventriculomegaly, corpus callosum agenesis, renal anomaly, duodenal atresia, and polyhydramnios. At birth, the patient needed respiratory support, and continuous positive airway pressure (CPAP) therapy was provided. Abdominal surgery was performed at day 1 due to suspicion of duodenal atresia, and an apple-peel anomaly was confirmed. After birth, bilateral renal hypoplasia was ascertained. She had bilateral microphthalmia (Figure 2) and sclerocornea. Brain MRI showed a cystic expansion of the cerebrospinal fluid (CSF). The CSF expansion was also in part secondary to reduced volume of the posterior part of the left cerebral hemisphere, chiefly due to marked hypoplasia of the left occipital lobe (compatible with colpocephaly), seemingly communicating with the left lateral ventricle (Figure 2). The cortex showed modest gyration compared to gestational age, which gave the impression of lissencephaly, and an underdeveloped corpus callosum (Figure 2). She died 26 days after birth.

Patient 2 was a 7-year-old female born to non-consanguineous healthy Australian parents, of Caucasian descent. Antenatally, there were several dilated loops of bowel with echogenic borders, observed throughout pregnancy from 19 weeks of gestation. She was born at 35 + 4 weeks gestation with weight 2600 g (50th centile), length 46 cm (50th centile), and OFC 31 cm (3rd centile). The patient had jejunal and ileal atresia, which was surgically corrected at day 1. The jejunal and ileal atresia did not have an apple-peel appearance at barium enema. At 7 months, the brain ultrasound examination, although limited due to a small anterior fontanelle, did not reveal anomalies. There was no evidence for intracranial hemorrhage, mass or white matter abnormality; the ventricular system was of normal size and configuration; the visualized segment of the superior sagittal sinus was patent. At 15 months, weight was 15 kg (15th centile), length was 67 cm (3 cm < 3rd centile), and she was markedly microcephalic with OFC 40.5 cm (5 cm < 3rd centile). She had short palpebral fissures and sparse eyebrows, particularly at the medial aspects (Figure 1D). Her ophthalmic assessment revealed right Peters anomaly type 1. Ultrasound examination of the eye globes revealed no abnormalities, and clinically there was no esotropia. Renal ultrasound at age 29 months showed normal structure and the right kidney measured 5.7 cm and left kidney 5.4 cm (approximately 3rd centile). Her developmental assessment using *Griffiths Scales of Child Development*, 3rd Edition, was normal at the age of 34 months. At the age of 7 years, the patient had normal learning and received adequate reports from school for Maths and English.

Patient 3 was a 7-year-old male born to non-consanguineous healthy parents from Poland (Figure 1E). The delivery was at 39 weeks and the baby was small for gestational age with weight 1904 g (<3rd centile), length 46 cm (<3rd centile), and pronounced microcephaly with OFC 26 cm (6.5 cm < 3rd centile). He underwent two-stage bowel surgery to repair a type IIIb intestinal atresia on days 2 and 7. At 2 years of age, he continued to be microcephalic with OFC 36 cm (9.5 cm < 3rd centile). Brain MRI at the age of 3 years revealed colpocephaly. He had bilateral sclerocornea and bilateral renal hypoplasia and dysplasia. His developmental assessment with Polish Childhood Developmental Scale revealed low overall level of functioning, including very low skills in eye-motor coordination, comparing shapes or objects, and speech. At 6.5 years of age, he learned to speak in full sentences, while being capable of pronouncing an adequate number of words or simple phrases. He was considered to have attention deficit hyperactivity disorder (ADHD) and developmental delay.

### 3.2. Genetic Analyses

Patient 1. Analysis of trio WES data identified a homozygous splice donor variant in *CENPF* (OMIM 600236), Chr1(GRCh37):g.214795625G>A, NM_016343.3:c.1068+1G>A, p.(Glu289Valfs*33). Sanger sequencing confirmed that the variant was homozygous in the patient and heterozygous in the parents (Figure 1A). This variant, ID rs1413067114, is reported in gnomAD (31) with an allele frequency of 4.32 × 10^−6^, only in the heterozygous state. The variant, located in *CENPF* intron 7, is in silico predicted to be pathogenic with a Combined Annotation Dependent Depletion (CADD) Phred score of 33 (32). Reverse transcriptase (rt)PCR amplification from leukocyte RNA from the parents followed by Sanger sequencing documented aberrant splicing leading to skipping of *CENPF* exon 7 (203 bp) (Figure 1B,C), predicted to result in p.(Glu289Valfs*33). RNA from the patient was not available. The *CENPF* variant was concluded to be pathogenic and causative of Strømme syndrome in patient 1.

Single gene sequencing of patient 2 revealed two compound heterozygous variants in *CENPF* (Figure 1D). The variants were the following: *CENPF*: Chr1 (GRCh37): g.214818833dup, NM_016343.3:c.5920dup; p.(Thr1974Asnfs*9), ID rs757531591, not reported in gnomAD [28]; and CADD Phred 24 [29]; and Chr1(GRCh37): g.214832221del, NM_016343.3: c.8991del; p.(Ser2998Alafs*23), ID rs752455487, reported in gnomAD [28] with an allele frequency of 2.84 × 10^−5^ in heterozygosity, CADD Phred 33 [29]. The variant p.(Thr1974Asnfs*9) has been previously identified in a patient with Strømme syndrome [1], while p.(Ser2998Alafs*23) is a novel variant. Parental testing confirmed that the parents were carriers. The two *CENPF* variants were classified as pathogenic and considered causative of the Strømme syndrome.

**Figure 3:**
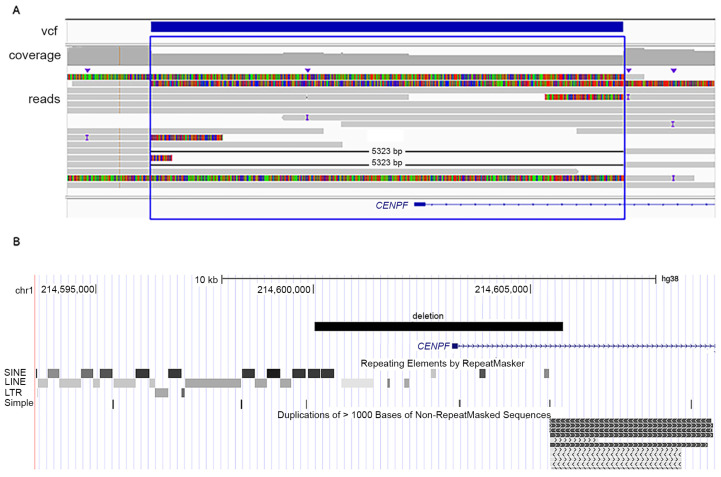
(**A**) LR-WGS data of patient 3 showing a 5323 bp deletion in *CENPF*: Chr1(GRCh38) g.214600232-214605555, covering exon 1 and part of intron 1. The blue bar indicates the deletion as annotated in the vcf file. Soft clips (colorful bars) identify reads that span the deletion and only partially align to the reference genome. The deletion was present in a heterozygous state. Data are shown in IGV [27]. (**B**) Screenshot from UCSC genome browser showing the genomic location of the heterozygous 5323 bp deletion overlapping *CENPF* exon 1 in patient 3. The proximal breakpoint of the deletion is embedded in a region enriched of repetitive elements. The distal breakpoint of the deletion overlaps segmental duplications.

**Figure 4:**
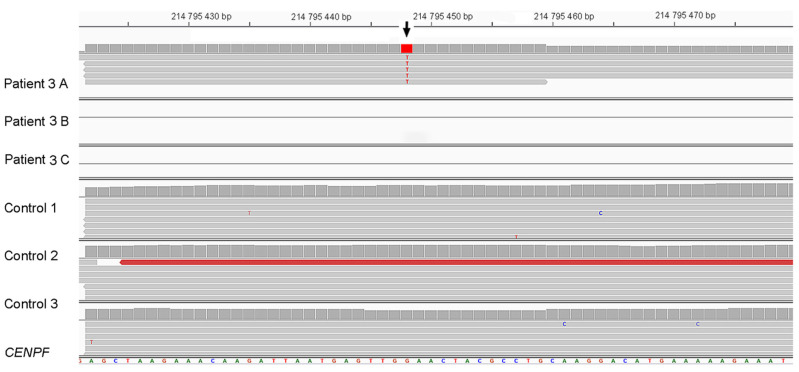
RNA-seq data of patient 3 (biological triplicates, A–C) and three controls (Control 1–3). In patient 3, only one of the three replicates detected sequence reads from this region of *CENPF*, which indicated monoallelelic expression as all five reads contained c.892G>T. Data are shown in IGV [27].

Patient 3. Analysis of the WES data of the patient revealed a heterozygous pathogenic variant in *CENPF*: Chr1(GRCh37):g.214795448G>T, NM_016343.3:c.892G>T, p.(Glu298Ter) (Figure 1E), with CADD Phred score of 43 (32), and allele frequency of 7.97 × 10^−6^ and only in heterozygous state in gnomAD database [28] (ID rs749662088). The clinical phenotype of patient 3 was compatible with Strømme syndrome, which has autosomal recessive inheritance. However, despite an overall read depth of 30× for *CENPF* in the WES data, a second putative pathogenic variant in this gene remained elusive. Therefore, we performed LR-WGS to study structural variants possibly affecting *CENPF*. The LR-WGS generated 2,446,247 HiFi reads, corresponding to 36.68 Gb HiFi yield and 15 kb mean HiFI read length. Quality metrics of the LR-WGS are available in Table S1. LR-WGS data analysis identified a 5323 bp deletion Chr1(GRCh38): g.214600232-214605555, covering exon 1 and into intron 1 of *CENPF*. The deletion was heterozygous in the patient (Figure 3A). The proximal breakpoint of the deletion mapped in an Alu element, part of a 13 kb cluster of repeated elements, while the distal breakpoint was part of a large segmental duplication (Figure 3B). The deletion was not reported in the following databases: gnomAD SVs v2.1, NCBI ClinVar and dbVar, Database of Genomic Variants (DGV), Decipher CNVs. We then performed RNA-seq in fibroblasts from patient 3 and three controls (quality metrics in Table S2 and transcript levels in Table S3). The data analysis revealed monoallelic expression of the *CENPF* c.892G>T; p.(Glu298Ter) in patient cells (Figure 4). The total read count for *CENPF* in the RNA-seq data was 95 in patient 3 versus 4314 in the controls (*p* < 0.0001) (Table S4), and only the transcript with the *CENPF* c.892G>T variant was detected (five reads, Figure 4). This significantly reduced *CENPF* transcript level confirmed that the 5323 bp heterozygous deletion in *CENPF*, overlapping exon 1, behaved as a null allele. This result also indicated that the transcript with the *CENPF* c.892G>T variant is likely undergoing nonsense-mediated mRNA decay.

In an attempt to assess the inheritance of the intergenic deletion in patient 3, eight different sets of primers were designed (~800 bp each) covering the entire region deleted in the patient. All PCR products from patient 3 and his parents were used to prepare libraries utilizing the NEXTERA XT kit (Illumina). Sequencing of the libraries on the NovaSeq platform confirmed the presence of the c.892G>T variant, which was inherited from the father. However, patient 3 and both parents were homozygous for all SNPs in the sequenced amplicons, thus preventing testing of the presence of the deletion in the parents. In summary, the two *CENPF* variants in patient 3 were classified as pathogenic and considered causative of the Strømme syndrome.

## 4. Discussion

Strømme syndrome is an ultra-rare primary ciliopathy with only 18 families previously reported with documented bi-allelic pathogenic variants in *CENPF* [1,2,3,4,5,6,7,8,9,10,11,12,13,14,15]. In addition, patients with phenotypes compatible with Strømme syndrome, but without identified pathogenic variants in *CENPF*, have been reported before the genetic basis was discovered [19,30,31,32,33,34]. Because of mid-gestation lethality [15], elective abortion, and neonatal death [9], the true incidence of Strømme syndrome may be underestimated. Most patients with a molecular diagnosis carry null alleles, but more recently five independent patients with missense variants in *CENPF* and Strømme syndrome were reported, possibly with a milder phenotype, as they did not present with intestinal atresia and eye anomalies [3,4,7,14]. Cappuccio et al. [7] documented the pathogenicity of a *CENPF* missense variant in cells from their patient 1, as they measured decreased ciliary length and reduced fraction of ciliated cells. The clinical manifestations in patient 3 in the present study were compatible with a diagnosis of Strømme syndrome, and the WES analysis identified a heterozygous pathogenic variant in *CENPF*. However, the second variant was identified only after implementation of a LR-WGS approach, which allowed us to detect a structural variant on the other *CENPF* allele, and thus concluding the molecular diagnosis of Strømme syndrome. In fact, integration of LR-WGS approaches, combined with improved bioinformatic pipelines, has started to unravel the important contribution of structural variants in Mendelian diseases [35,36]. Our finding expands the spectrum of genetic defects causing Strømme syndrome, reporting for the first time a 5323 bp intragenic deletion in *CENPF* overlapping exon 1 and part of intron 1, identified in trans with a frameshift variant in patient 3. Both breakpoints of the *CENPF* deletion mapped to genomic regions enriched of repetitive elements (proximal breakpoint) or segmental duplication (distal breakpoint), conceivably predisposing the region flanking *CENPF* exon 1 to genomic rearrangements. Therefore, structural variants affecting *CENPF* exon 1 should be carefully analyzed in patients with a clinical diagnosis of Strømme syndrome but lacking a molecular diagnosis.

Ciliopathies are considered Mendelian disorders, but the extensive phenotypic heterogeneity has been ascribed to more complex underlying genetic defects, with a contribution from digenic inheritance and modifier alleles, as seen for example in Bardet–Biedl syndrome (OMIM 209900) [37]. However, in Strømme syndrome, more complex genetic inheritance and its impact on the phenotypic variability has not previously been documented. In line with the extensive phenotypic heterogeneity documented in many ciliopathies [38] and in Strømme syndrome [11], the severity of the disease varies remarkably as illustrated in this report, although all have *CENPF* null alleles. In fact, in patient 1 the cerebral, ocular, intestinal, and renal anomalies coincided with neonatal lethality. Patients 2 and 3 had multi-organ anomalies and their clinical manifestations are typical of Strømme syndrome: microcephaly, ocular anomalies, and intestinal atresia, but overall they are less severely affected, possibly also because severe brain malformation was not present. In line with this, patient 2 had not yet manifested cognitive delay. Normal intellect in Strømme syndrome has so far been reported only in one adult patient [10] and one patient has been described with below average IQ with score 83 in a pre-school cognitive development assessment [2].

All three patients in the current report had intestinal atresia, which was present in the first patients reported with Strømme syndrome [1]. Intestinal atresia with apple-peel malformation consists of a proximal jejunum ending in a blind pouch and a distal small bowel wrapped around its vascular supply in a spiral fashion. It is a rare form of bowel atresia estimated to affect approximately 1/50,000 babies [39] and its documentation in patients was intended as suggestive of Strømme syndrome. However, recently patients with *CENPF* null alleles and without apple-peel atresia have been described [13] or with intestinal atresia not of apple-peel type, like in patient 2.

As proper functioning of primary cilia is essential to kidney organogenesis and maintenance, a spectrum of renal diseases is frequently observed in many ciliopathies, for example autosomal dominant and recessive polycystic kidney disease, Bardet–Biedl syndrome, Joubert syndrome, Meckel–Gruber syndrome, Nephronophthisis, and Senior–Loken syndrome [40]. The kidneys are often affected also in Strømme syndrome [11]. Patient 1 and 3 had hypoplastic kidneys, while patient 2 did not have a renal pathology, although the kidneys sizes were at the low end of the normal range. A young adult with Strømme syndrome presenting with acute renal failure has been reported in [8]. This may suggest that regular follow up of renal function in patients with Strømme syndrome should be part of the clinical disease management. Currently, clinical practice guidelines for Strømme syndrome have not been defined [11], possibly due to low disease prevalence, but also to phenotypic variability, which may require tailor-made clinical follow-up.

## 5. Conclusions

In summary, we report three unrelated patients with Strømme syndrome an ultra-rare primary ciliopathy. All three patients had bi-allelic null alleles, but despite this, clinical manifestations and severity were varied. The genetic basis of such clinical variation at the moment is not known and deserves further work. We also identified the first intragenic deletion in a complex genomic region of *CENPF*. We suggest that this region should be carefully analyzed in patients with a phenotype compatible with Strømme syndrome, but without pathogenic SNVs/indels in the gene.

## Figures and Tables

**Table 1:** Genetic and clinical findings of patients 1–3.

Patients	Patient 1	Patient 2	Patient 3
Variants in *CENPF*	c.1068+1G>A; p.Glu289Valfs*33 homozygous	c.5920dup; p.Thr1974Asnfs*9/ c.8991del; p.Ser2998Alafs*23	c.892G>T; p.Glu298Ter/5 kb intragenic deletion
Country of origin	Lithuania	Australia	Poland
Age at last examination (gender)	Neonatal, died at 26 days (female)	7 years (female)	7 years (male)
Perinatal history	Preterm (34 weeks), SGA	Born at term	Born at term, SGA
OFC at birth	25.8 cm, 3 cm < 3rd centile	31 cm, 2 cm < 3rd centile	26 cm, 6.5 cm < 3rd centile
Development	Not assessed	Normal	Delayed, especially speech; ADHD
Brain MRI examination	Brain cyst (colpocephaly), corpus callosum hypoplasia, primitive gyrosulcal pattern	Not performed. Only ultrasound examination	Colpocephaly
Ocular examination	Microphthalmia, sclerocornea	Peters anomaly type 1	Microphthalmia, sclerocornea
Gastrointestinal anomalies	Duodenal atresia apple-peel type	Jejunal and ileal atresia	Jejunal atresia apple-peel type IIIb intestinal atresia
Kidneys	Hypoplasia	Borderline size 3rd centile	Hypoplasia, dysplasia

OFC: occipitofrontal circumference; SGA: small for gestational age.

## Data Availability

The data will be available upon request. Distribution of sensitive data may be subject to restrictions.

## Supplementary Materials

The following supporting information can be downloaded at: https://www.mdpi.com/article/10.3390/genes14111985/s1, Table S1: LR-WGS sequencing quality metrics; Table S2: Quality metrics of RNA-seq from skin-derived fibroblasts of patient 3 and controls; Table S3: Transcript levels in RNA-seq data of patient 3; Table S4: Normalized gene read counts of *CENPF* in RNA-seq data of patient 3 and three controls.
